# Spatial distribution and factors associated with modern contraceptive use among women of reproductive age in Nigeria: A multilevel analysis

**DOI:** 10.1371/journal.pone.0258844

**Published:** 2021-12-08

**Authors:** Obasanjo Afolabi Bolarinwa, Zemenu Tadesse Tessema, James Boadu Frimpong, Abdul-Aziz Seidu, Bright Opoku Ahinkorah

**Affiliations:** 1 Department of Public Health Medicine, School of Nursing and Public Health, University of KwaZulu-Natal, Durban, South Africa; 2 Obaxlove Consult, Lagos, Nigeria; 3 Department of Epidemiology and Biostatistics, Institute of Public Health, College of Medicine and Health Sciences, University of Gondar, Gondar, Ethiopia; 4 Department of Health, Physical Education, and Recreation, University of Cape Coast, Cape Coast, Ghana; 5 Department of Population and Health, University of Cape Coast, Cape Coast, Ghana; 6 College of Public Health, Medical and Veterinary Sciences, James Cook University, Townsville, Australia; 7 School of Public Health, University of Technology Sydney, Sydney, NSW, Australia; University of Miami, UNITED STATES

## Abstract

**Background:**

Evidence suggests that in countries with high fertility and fecundity rates, such as Nigeria, the promotion of modern contraceptive use prevents approximately 32% and 10% of maternal and child mortality, respectively. Therefore, this study aimed to assess the spatial distribution of modern contraceptive use and its predictors among women of reproductive age in Nigeria.

**Methods:**

The study employed a cross-sectional analysis of population-based data involving 24,281 women of reproductive age in Nigeria. The study adopted both multilevel and spatial analyses to identify the predictors of modern contraceptive use and its spatial clustering among women in Nigeria.

**Results:**

Modern contraceptive use among the study population in Nigeria ranged from 0% to 75%, with regional variations. The spatial analysis showed that areas with a low proportion of modern contraceptive use were Sokoto, Yobe, Borno, Katsina, Zamfara, Kebbi, Niger, Taraba and Delta. Areas with a high proportion of modern contraceptive use were Lagos, Oyo, Osun, Ekiti, Federal capital territory, Plateau, Adamawa, Imo, and Bayelsa. The multilevel analysis revealed that at the individual level, women with secondary/higher education, women from the Yoruba ethnic group, those who had four children and above, and those exposed to mass media had higher odds of using modern contraceptives. On the other hand, women who were 35 years and above, those who were married, and women who were practicing Islam were less likely to use modern contraceptives. At the household/community level, women from the richest households, those residing in communities with medium knowledge of modern contraceptive methods, and women residing in communities with a high literacy level were more likely to use modern contraceptives.

**Conclusion:**

There were major variations in the use of modern contraception across various regions in Nigeria. As a result, areas with low contraceptive rates should be given the most deserving attention by promoting contraceptive education and use as well as considering significant factors at the individual and household/community levels.

## Background

Over the years, Nigeria has emerged as one of the countries with the fastest-growing population globally due to the low utilization of contraceptives [1]. It is estimated that Nigeria’s population exceeds 170 million, and more than 400,000 Nigerian women’s lives are lost to childbirth and associated intricacies from unintended pregnancies every year [2, 3]. Research indicates that in countries with high fertility and fecundity rate, such as Nigeria, the promotion of modern contraceptive use prevents approximately 32% and 10% of maternal and child mortality, respectively [4]. Besides that, modern contraceptive utilization plays a contributory role in achieving universal primary schooling, empowering females, and lessening poverty and hunger [5]. Again, modern contraceptives prevent unplanned pregnancies and unsafe abortions [6, 7].

Notwithstanding the well-known importance of modern contraceptives, access to and utilization of the contraceptives differ among populations worldwide [4]. Research points out that compared to women in low-and middle-income countries like Nigeria, those in high income countries have improved access to and use of modern contraceptives [4]. A study reported that though the global prevalence of unplanned pregnancy from 2010 to 2014 stood at 44%, the prevalence was even higher in low-and middle-income countries [8]. Additionally, it is suggested that higher levels of unmet need for contraception are probably the cause of heightened rates of unplanned pregnancies in low-and middle-income countries like Nigeria [4, 9, 10].

In Nigeria, most women, especially those in rural settlements, have numerous challenges accessing and utilizing modern contraceptives [11]. For example, some socio-cultural beliefs may hinder women’s uptake of modern contraceptives even if they would willingly do so [11, 12]. Factors such as age, residence, region, educational level, partner’s educational level, household wealth, employment status, number of living children, and religion have been identified in previous studies as factors that influence the use of modern contraceptives among women of reproductive age [4, 13–16].

To the best of our knowledge, no study has assessed the spatial distribution of modern contraceptive use and the factors associated with modern contraceptive use among women of reproductive age in Nigeria using current nationally representative data. This makes it difficult to formulate and implement policies and interventions specific to Nigeria’s various regions or clusters. This presents a void in the extant literature which this study seeks to address. Therefore, this study assessed the spatial distribution of modern contraceptive use and the factors that influence its use among women of reproductive age in Nigeria, using the recent Nigeria Demographic and Health Survey (NDHS) data conducted in 2018. The study’s findings could help direct policies and help reduce unplanned pregnancies and unsafe abortions in Nigeria.

## Methods and materials

### Data source

This is a cross-sectional analysis of a population-based study from the 2018 NDHS. The NDHS is a nationally representative survey used to gather sociodemographic and other health-related indicators such as modern contraceptive use [17]. The survey collected data from 36 administrative units and the Federal Capital Territory (FCT) through a two-stage sampling process. The samples which were randomly selected from clusters or enumeration areas (EAs) served as the survey’s primary sampling unit. The 2018 survey drew a total of 41,821 women aged 15 to 49. From this number, 24,281 women of reproductive age who had complete information on modern contraceptive use and all the variables of interest were included in this study. The sampling, pretesting, and the general methodology of the 2018 NDHS has been published elsewhere [18]. The study followed the strengthening of the reporting of observational studies in Epidemiology in writing this manuscript [19]. The dataset is freely accessible for download via https://dhsprogram.com/data/available-datasets.cfm.

### Variables

#### Dependent variable

The dependent variable was modern contraceptive utilization. If a woman uses at least one of the following methods: female sterilization, male sterilization, intrauterine device [20], injectable, implants, pills, male condom, female condom, emergency contraception, and standard days method, she is considered a modern contraceptive user. Non-users, on the other hand, are those who rely on traditional methods like the rhythm method, lactational amenorrhea method, and withdrawal, or if she has never used contraception at all [4, 21–23].

#### Independent variables

Individual and household/community level factors were considered as independent variables in this study. The individual-level factors included age (15–24, 25–34, 35+), educational level (no education, primary, secondary/higher), marital status (never married, married, cohabiting, separated/divorced/widowed), religion (Christianity, Islam, traditionalist/others), working status (not working, working), ethnicity (Hausa, Yoruba, Igbo, others), parity (0, 1–3, 4+), Media exposure (yes, no). The household/community level variables included in the study were wealth index (poorest, poorer, middle, richer, richest), region (Northcentral, Northeast, Northwest, Southeast, South-south, Southwest), sex of household head (male, female), community knowledge of modern contraceptives (low, medium, high), community literacy level (low, medium, high), community socioeconomic status (low, medium, high). All these variables were considered based on their theoretical and practical relevance to modern contraceptive usage and their availability in the 2018 NDHS dataset [4, 9, 21, 22, 24–26].

### Statistical analyses

The data were analyzed using both spatial and multilevel analysis.

### Spatial analysis

The weighted frequency of modern contraceptives with cluster number and geographic coordinate data were merged in Stata 14, and observations within seven clusters having longitude and latitude 0 were dropped. Data was then exported to excel with then imported to ArcGIS 10.7 for spatial analysis.

### Spatial autocorrelation analysis

The spatial autocorrelation (Global Moran’s I) statistic measures how dispersed, clustered, or randomly distributed in modern contraceptive use among women of reproductive age in Nigeria. Moran’s I is a spatial statistic used to measure spatial autocorrelation by taking the entire data set and produce a single output value that ranges from -1 to +1. Moran’s I Values close to −1 indicate dispersed modern contraceptive use, while I close to +1 indicate modern contraceptive use clustered and modern contraceptive use distributed randomly if I value is zero. A statistically significant Moran’s I (p < 0.05) leads to the failure to reject the alternative hypothesis and rejection of the null hypothesis (modern contraceptive use is randomly distributed) and indicates the presence of spatial autocorrelation.

### Hot spot analysis (Getis-OrdGi* statistic)

Getis-OrdGi* statistics were computed to measure how spatial autocorrelation varies in Nigeria by calculating the GI* statistics for each area. To compute and determine the statistical significance of clustering, the p-value is computed for significance using Z-score. Statistical output with low GI* means a “cold spot.” while high GI* indicates “hotspot”.

### Spatial interpolation

It is very expensive and laborious to collect reliable data in all areas of the country to know the burden of a certain event. Therefore, interpolation was used to predict part of a certain area using observed data. The spatial interpolation technique predicts modern contraceptive use on the un-sampled areas in the country based on sampled EAs from DHS. The ordinary Kriging spatial interpolation method was used for this study to predict modern contraceptive use in unobserved areas of Nigeria. For this study, the ordinary Kriging method was used to estimate the burden of modern contraceptive use in unsampled areas [27].

### Multilevel analysis

A two-level multilevel binary logistic regression models were fitted to evaluate the individual and household/community level factors linked to modern contraceptive use. Women were nested within households, and households were nested within clusters. Clusters were considered as random effects to account for the unexplained variability at the community level. Four models were fitted. We began by fitting the empty model, model I, which contained no predictors (random intercept). Model II only included individual-level variables, model III only included household/community level l variables, and model IV included both individual-level and household/community level variables. The adjusted odds ratios (aORs) and related 95% confidence intervals (CIs) were provided for all models. These models were fitted by a Stata command “melogit” to identify predictors with the outcome variable. The log-likelihood ratio (LLR), Akaike Information Criteria (AIC) measure, and Schwarz’s Bayesian Information Criteria (BIC) were used to compare models. The best fit model has the highest log-likelihood and the lowest AIC [4, 28]. The women population sample weight (v005/1,000,000) was used in all analyses to account for over-and under-sampling, while the svy command was used to account for the complex survey design and generalizability of the results. All the analyses were carried out using Stata version 16.0 (Stata Corporation, College Station, TX, USA).

## Result

### Socio-demographic characteristics

A total of 24,281 women of reproductive age were included in the study. Out of this number, 39.53% were between the ages of 25–34, 44.78% had no formal education, 91.03% were married, and 60.18% practiced Islam. Also, 43.85% of the respondents belonged to the Hausa ethnic group, 63.70% were exposed to mass media, 59.84% resided in rural areas, 20.79% were from the poorest households, 68.35% were from communities with medium knowledge of modern contraceptives, 34.97% were from communities with high literacy level, and 35.84% were from communities with high socioeconomic status (Table 1).

**Table 1:** Individual and household/community level characteristics of reproductive age women in Nigeria (n = 24,281).

Variable	Weighted Frequency	Percentage
Individual level		
**Age of respondent**		
15–24	5,868	24.17
25–34	9,598	39.53
35 & above	8,815	36.30
**Level of education**		
No Education	10,872	44.78
Primary	3,466	14.27
Secondary & above	9,943	40.95
**Marital status**		
Never married	1,212	4.99
Married	22,103	91.03
Cohabitating	666	2.74
Separated/divorced/widowed	299	1.23
**Religious affiliation**		
Christianity	9,395	38.69
Islam	14,766	60.81
Traditionalist & others	120	0.49
**Working status**		
Not working	7,355	30.29
Working	16,925	69.71
**Ethnicity**		
Hausa	10,647	43.85
Yoruba	3,243	13.36
Igbo	2,790	11.49
Others	7,600	31.30
**Parity**		
0	3,051	12.56
1–3	11,122	45.81
4 & above	10,108	41.63
**Exposure to media**		
No	8,813	36.30
Yes	15,467	63.70
**Household/community level**		
**Place of residence**		
Urban	9,751	40.16
Rural	14,529	59.84
**Wealth index**		
Poorest	5,047	20.79
Poorer	5,190	21.38
Middle	4,540	18.70
Richer	4,672	19.24
Richest	4,830	19.89
**Region**		
North Central	3,097	12.76
North East	4,345	17.89
North West	8,612	35.47
South East	1,978	8.15
South South	2,490	10.26
South West	3,759	15.48
**Sex of household head**		
Male	22,385	92.19
Female	1,896	7.81
**Community modern contraceptive method knowledge**		
Low	7,685	31.65
Medium	16,596	68.35
**Community literacy level**		
Low	8,053	33.17
Medium	7,736	31.86
High	8,491	34.97
**Community socioeconomic status**		
Low	13,342	54.95
Medium	2,238	9.22
High	8,701	35.84

### Spatial analysis results

#### Spatial distribution of modern contraceptive use

**Fig 1:**
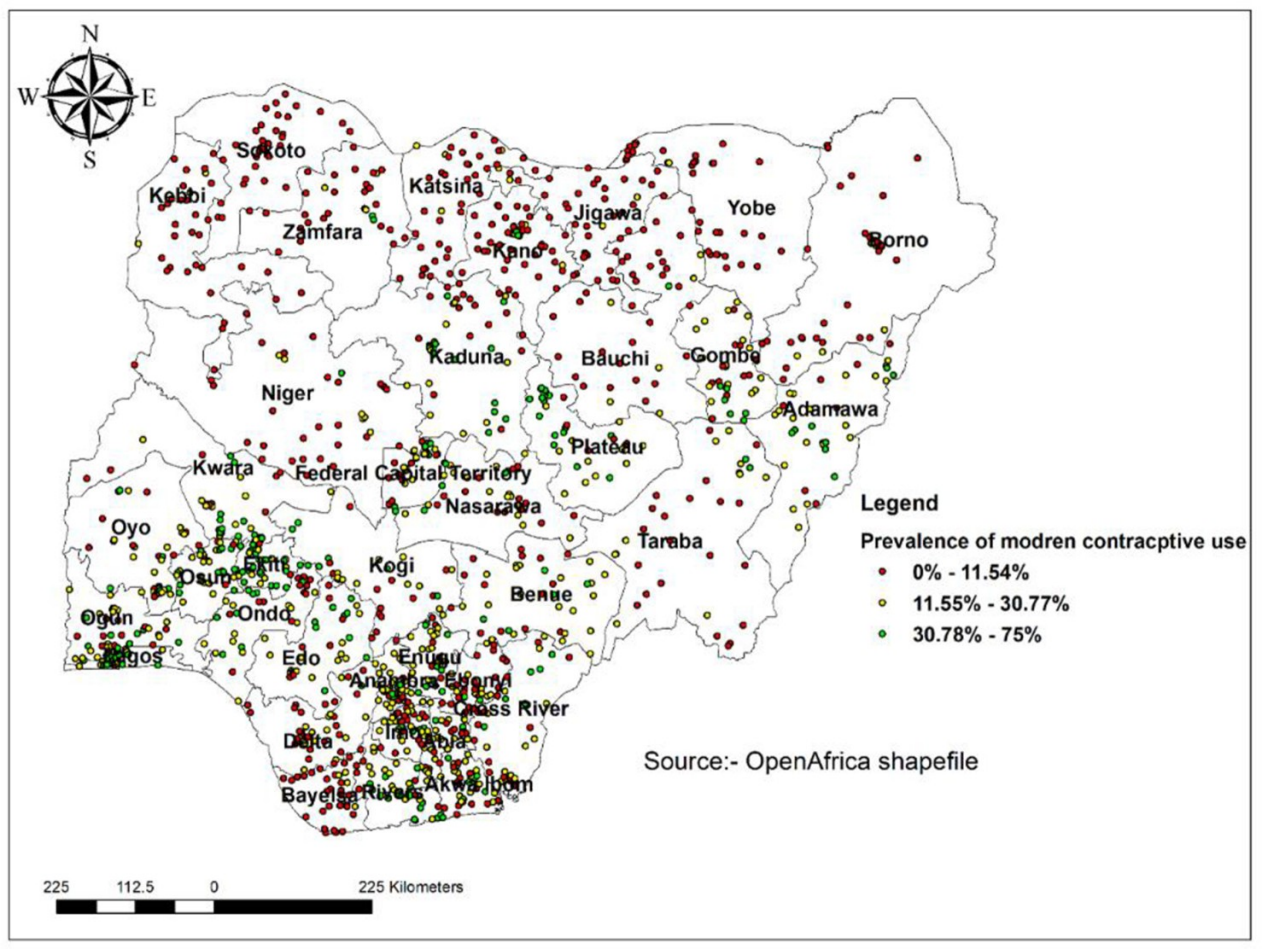
Spatial distribution of modern contraceptive use in Nigeria, 2018.

A total of 1,388 clusters were considered for the spatial analysis of modern contraceptive use in Nigeria. Each point on the map represents one enumeration area with the proportion of modern contraceptive use cases in each cluster. The red color indicates areas with low proportions of contraceptive use which ranged from 0–12%, and the blue color indicates EAs with a high proportion of modern contraceptive use ranging from 32–75%. The spatial distribution of modern contraceptive use in this study showed that a higher proportion of modern contraceptive use was located in the Southern part of Nigeria. A low proportion of modern contraceptive use was located in the Northern part of Nigeria (Fig 1).

#### Spatial autocorrelation modern contraceptive use

**Fig 2:**
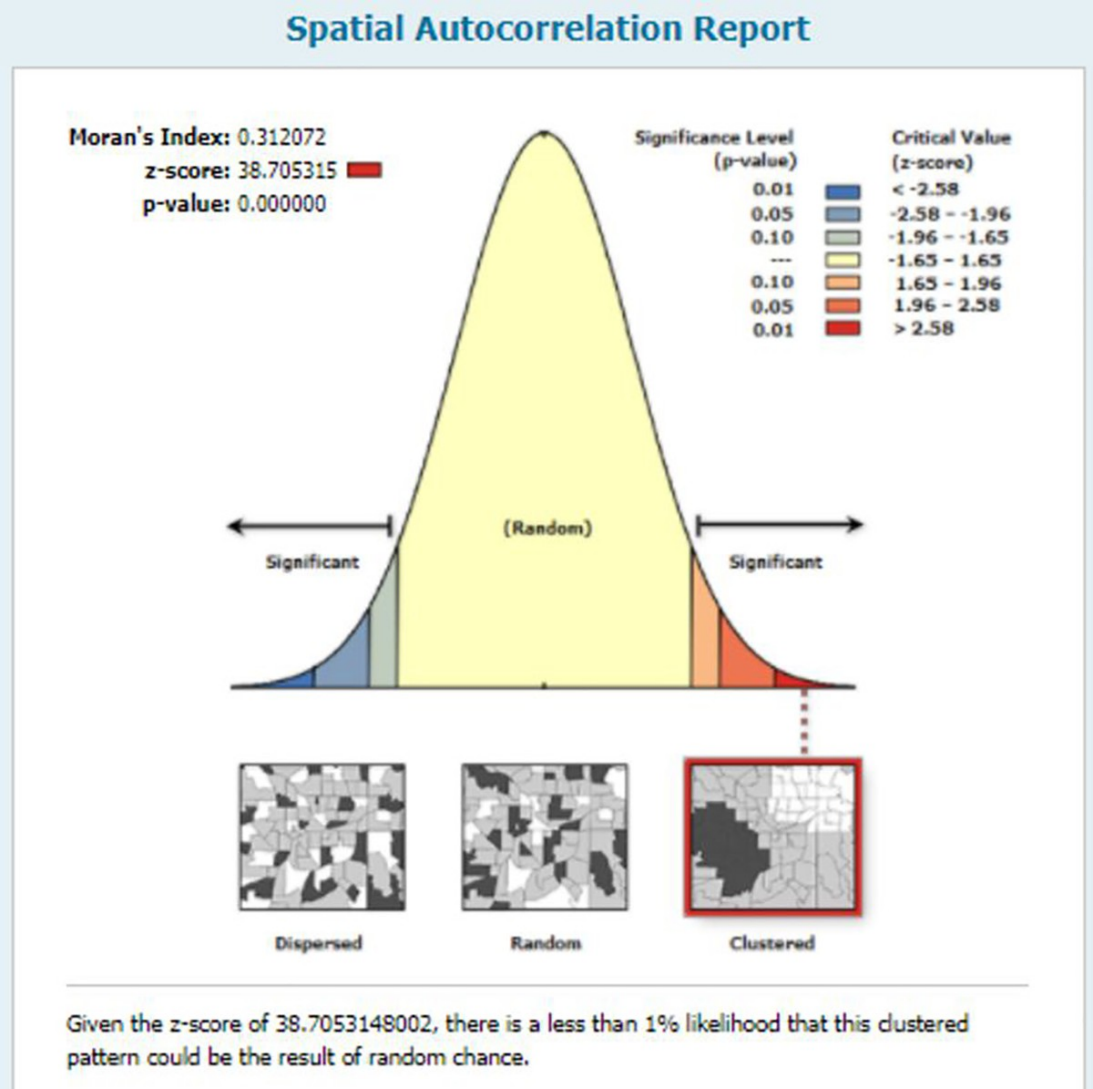
Spatial autocorrelation of modern contractive use in Nigeria, 2018.

The spatial autocorrelation result shows whether modern contraceptive use in Nigeria is dispersed, clustered, or random throughout the country. The finding of the spatial autocorrelation analysis revealed a clustering effect in modern contraceptive use across the country. The clustered patterns (on the right sides red box) showed a clustering effect on modern contraceptive use in Nigeria. The outputs have automatically generated keys on the right and left sides of each panel. The z-score of 38.70 (p-value = <0.001) indicated that there is less than 1% likelihood that this clustered pattern could be the result of random chance. The bright red and blue colors to the end tails indicate an increased significance level (Fig 2).

### The hotspot analysis result

**Fig 3:**
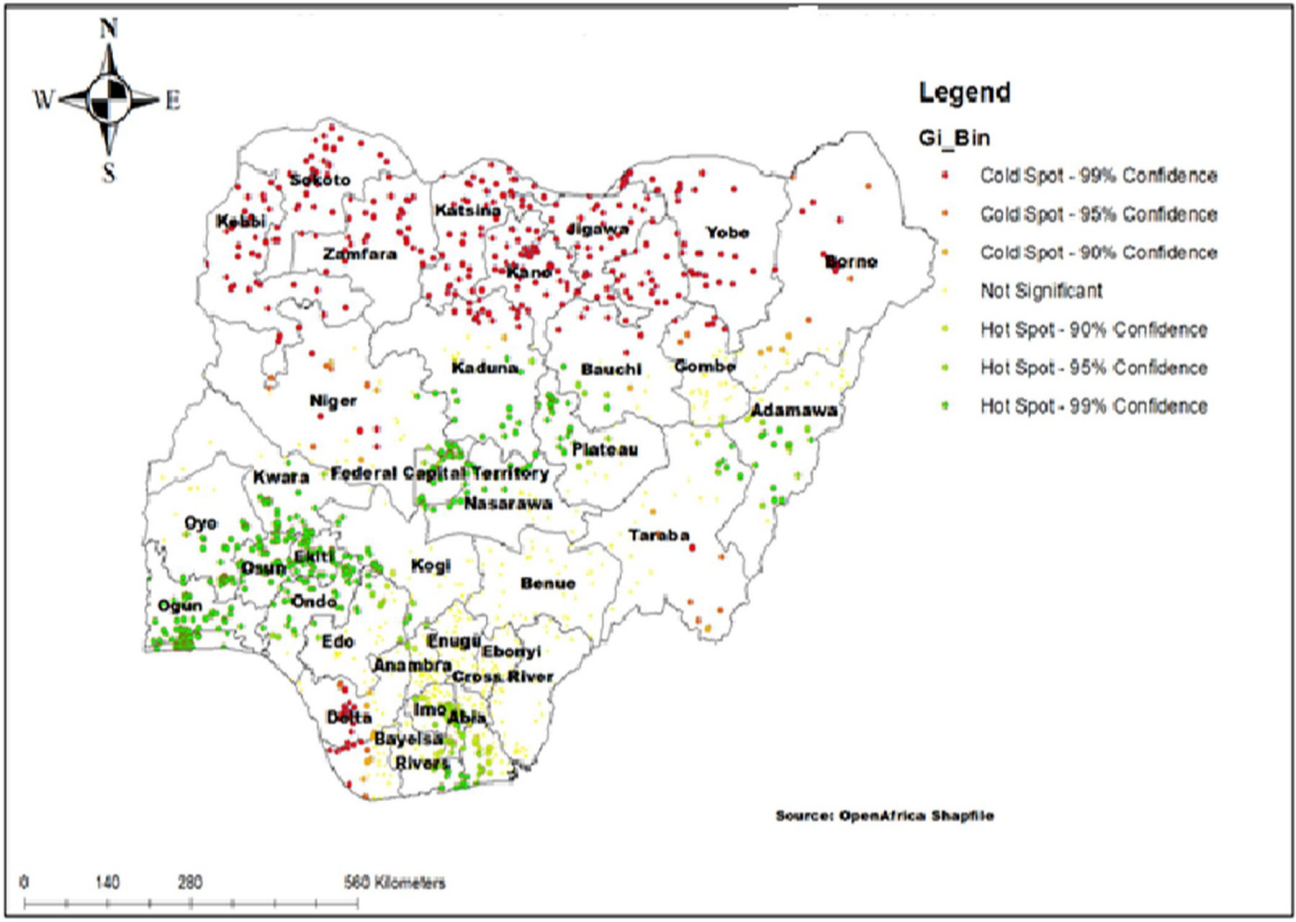
Hotspot analysis of modern contraceptive use in Nigeria, 2018.

The hotspot analysis result reveals the high proportion (hotspot) and low proportion (cold spot) areas of modern contraceptive use in Nigeria. In the cold spot areas (low proportion of modern contraceptive use), the red colors were located in Sokoto, Yobe, Borno, Katsina, Zamfara, Kebbi, Niger, Delta and Taraba states. The hotspot (high proportion of modern contraceptive use) areas were located in Lagos, Oyo, Osun, Ekiti, Federal capital territory, Plateau, Adamawa, Imo, and Bayelsa states (Fig 3).

### Spatial interpolation or prediction

**Fig 4:**
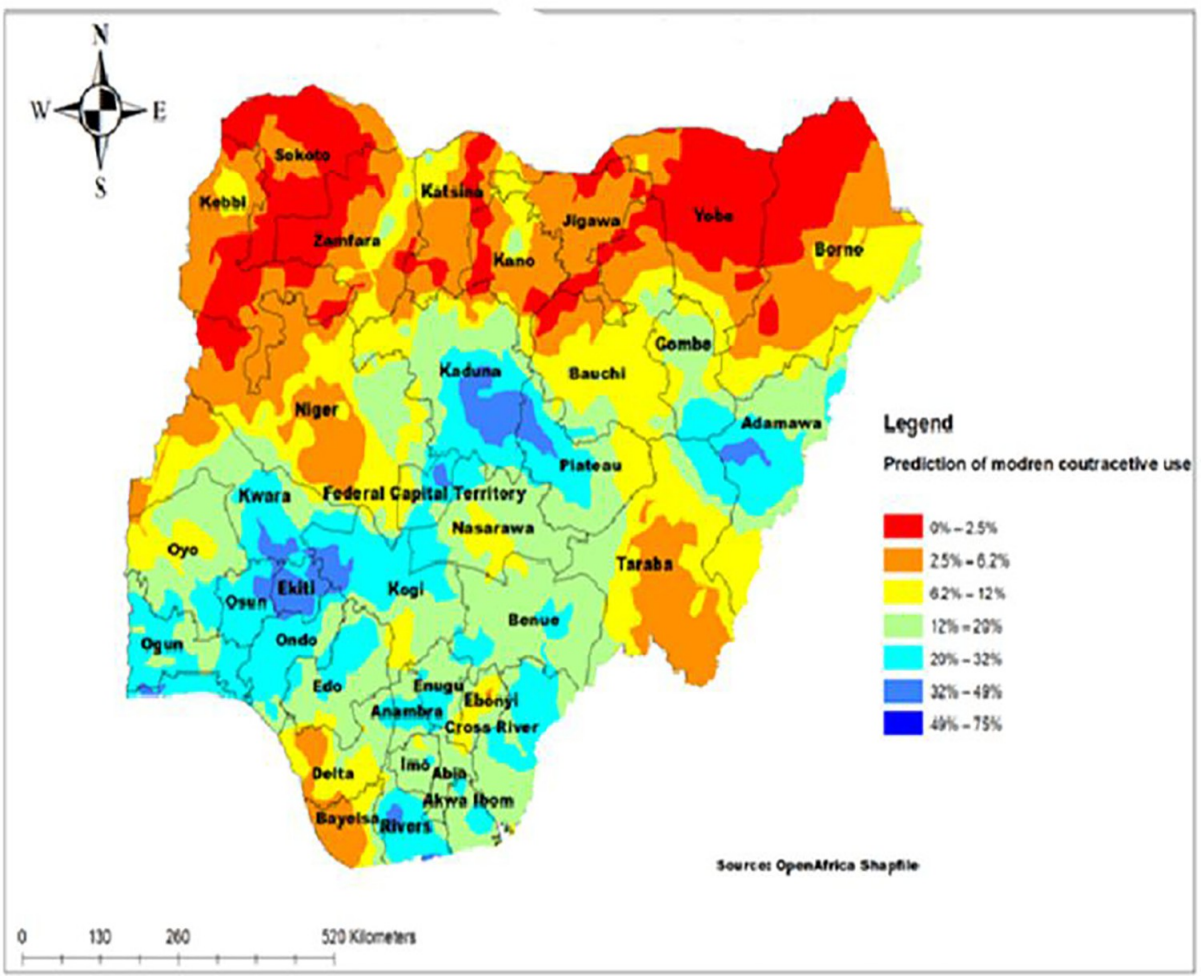
The prediction of modern contraceptive use in Nigeria, 2018.

The spatial interpolation technique shows the predicted proportion of modern contraceptive use for unsampled areas based on the sampled area. The ordinary Kriging method was used in describing the area map. If the area color changed from green to red, this means that the predicted modern contraceptive use decreases over the area. The red color indicates the predicted low utilization of modern contraceptive use in the country. According to the prediction result, a low proportion of modern contraceptive use is located in Sokoto, Yobe, Zamfara, Borno, and Kebbi. The blue color prediction indicated that a high proportion of modern contraceptive use in the country was located in Kaduna, Adamawa, Ekiti, Rivers, and Federal Capital Territory (Fig 4).

### Multi-level fixed effects (measures of associations) results

At the individual-level factors, the likelihood of using modern contraceptives among women of reproductive age was high among those who had secondary education and above [aOR = 2.15; 95% (CI = 1.85–2.50)], women who were from Yoruba ethnic group [aOR = 1.67; 95% (CI = 1.30–2.50)], those who had four children and above [aOR = 7.49; 95% (CI = 5.94–9.44)], and those who were exposed to mass media [aOR = 1.28; 95% (CI = 1.13–1.44)], compared to women who had no formal education, those who were from Hausa ethnic group, women with no children and those who were not exposed to mass media. Women who were 35 years and above [aOR = 0.79; 95% (CI = 0.68–0.92)], those who were married [aOR = 0.23; 95% (CI = 0.18–0.29)] and those practicing Islam [aOR = 0.60; 95% (CI = 0.52–0.69)] were less likely to use modern contraceptive.

At the household/community level, women who were from the richest household [aOR = 1.49; 95% (CI = 1.17–1.90)], those residing in communities with medium knowledge of modern contraceptive method [aOR = 1.48; 95% (CI = 1.27–1.71)] and women residing in communities with the high level of literacy [aOR = 2.25; 95% (CI = 1.76–2.89)] were more likely to use modern contraceptive compared to women residing in the poorest household, those residing in communities with low knowledge of modern contraceptive method, and low literacy level. Those women residing in the northwest region [aOR = 0.67; 95% (CI = 0.53–0.69)] were less likely to use modern contraceptives.

### Random effects (measures of variations) results

The ICC value in Model 1 shows that 31% of the variation in modern contraceptive use among women of reproductive age in Nigeria was attributed to the variation between Intra-Class Correlation, i.e., (ICC = 0.31). The variation between-clusters decreased to 14% in Model II (individual level only). In the household/community-level only (Model III), the ICC remains 14%, while the ICC declined to 11% in the complete model with both the individual and household/community factors (Model III). This further reiterates that the variations in the likelihood of modern contraceptive use among women of reproductive age in Nigeria are attributed to the clustering variation in PSUs. The Akaike’s Information Criterion (AIC) and Schwarz’s Bayesian Information Criteria (BIC) values showed a successive reduction, which means a substantial improvement in each of the models over the previous model and affirms the goodness of Model IV developed in the analysis. Therefore, Model IV, the complete model with both the selected individual and household/community factors, was chosen for predicting the eventuality or occurrence of modern contraceptive use among women of reproductive age in Nigeria (Table 2).

**Table 2:** Multilevel logistic regression models for individual and household/community predictors of modern contraceptive use.

Variables	Model 1	Model II	Model III	Model IV
		aOR[95% CI]	aOR[95% CI]	aOR[95% CI]
**Fixed effects results**				
**Individual-level variables**				
**Age of respondent**				
15–24		RC		RC
25–34		1.08 [0.94–1.23]		0.99 [0.87–1.14]
35 & above		0.89 [0.76–1.03]		0.79**[0.68–0.92]
**Educational level**				
No Education		RC		RC
Primary		2.16***[1.86–2.52]		1.77***[1.52–2.07]
Secondary & above		3.02***[2.61–3.48]		2.15***[1.85–2.50]
**Marital status**				
Never married		RC		RC
Married		0.23***[0.18–0.28]		0.23***[0.18–0.29]
Cohabitating		0.26***[0.19–0.35]		0.27***[0.20–0.37]
Separated/divorced/widowed		0.41***[0.29–0.58]		0.43***[0.30–0.61]
**Religious affiliation**				
Christianity		RC		RC
Islam		0.62***[0.54–0.71]		0.60***[0.52–0.69]
Traditionalist & others		0.36**[0.18–0.72]		0.44*[0.22–0.89]
**Working status**				
No		RC		RC
Yes		1.09 [0.97–1.21]		1.09 [0.97–1.21]
**Ethnicity**				
Hausa		RC		RC
Yoruba		2.46***[2.03–2.99]		1.67***[1.30–2.15]
Igbo		1.11 [0.89–1.38]		1.09 [0.82–1.44]
Others		1.42***[1.20–1.68]		1.22*[1.01–1.47]
**Parity**				
0		RC		RC
1–3		3.22***[2.61–3.98]		3.32***[2.69–4.11]
4 & above		7.01***[5.58–8.82]		7.49***[5.94–9.44]
**Exposure to media**				
No		RC		RC
Yes		1.48***[1.32–1.67]		1.28***[1.13–1.44]
**Household/Community level**				
**Place of residence**				
Urban			RC	RC
Rural			0.95 [0.82–1.09]	0.90 [0.79–1.04]
**Wealth index**				
Poorest			RC	RC
Poorer			1.22*[1.01–1.47]	1.17 [0.97–1.41]
Middle			1.43***[1.18–1.74]	1.28*[1.05–1.56]
Richer			1.73***[1.40–2.14]	1.49***[1.19–1.85]
Richest			1.72***[1.36–2.17]	1.49**[1.17–1.90]
**Region**				
North Central			RC	RC
North East			0.73**[0.59–0.90]	1.01 [0.82–1.23]
North West			0.38***[0.31–0.46]	0.67**[0.53–0.84]
South East			0.49***[0.39–0.61]	0.42***[0.32–0.56]
South-South			0.59***[0.48–0.73]	0.47***[0.38–0.57]
South West			0.91 [0.74–1.11]	0.69**[0.55–0.87]
**Head of household**				
Male			RC	RC
Female			1.17*[1.03–1.34]	0.94 [0.81–1.10]
**Community modern contraceptive method knowledge**				
Low			RC	RC
Medium			1.72***[1.47–2.01]	1.48*** [1.27–1.71]
**Community literacy level**				
Low			RC	RC
Medium			2.60***[2.11–3.21]	1.65***[1.34–2.03]
High			4.22***[3.30–5.41]	2.25***[1.76–2.89]
**Community socioeconomic status**				
Low			RC	RC
Medium			0.93 [0.74–1.15]	0.98 [0.79–1.21]
High			1.04 [0.86–1.26]	1.16 [0.97–1.40]
**Random effects results**				
PSU Variance (95% CI)	1.46[1.27–1.66]	0.54[0.46–0.66]	0.55[0.46–0.66]	0.42[0.33–0.52]
ICC	0.31	0.14	0.14	0.11
LR Test	χ2 = 1435.98, p<0.001	χ2 = 353.52, p<0.001	χ2 = 378.95, p<0.001	χ2 = 148.30, p<0.001
Wald χ2	Reference	1233.18***	813.59***	1435.88***
**Model fitness**				
Log-likelihood	-8717.03	-8085.29	-8316.22	-7952.53
AIC	17438.05	16206.58	16668.45	15973.06
BIC	17454.21	16352.01	16813.88	16247.77
Number of clusters	1388	1388	1388	1388

Weighted NDHS, 2018.

Exponentiated coefficients; 95% confidence intervals in brackets; AOR = adjusted Odds Ratios; CI = Confidence Interval; RC = Reference Category.

*p< 0.05

**p< 0.01

***p< 0.001.

ICC = Intra-Class Correlation; BIC = Schwarz’s Bayesian Information Criteria; AIC = Akaike’s Information Criterion; LR Test = Likelihood ratio Test; PSU = Primary Sampling Unit.

Model 0 is the null model, without any explanatory variable at the baseline model.

Model I is adjusted for individual-level variables (Age of respondent, media exposure, educational level, marital status, ethnicity, currently working, religious affiliation, and parity).

Model II is adjusted for household/community level variables (Place of residence, wealth index, region, sex of household head, community modern contraceptive method knowledge, community literacy level, community socioeconomic status).

Model III is the final model adjusted for individual and household/community level variables.

## Discussion

The study assessed the spatial distribution of modern contraceptive use and the factors that influence its use among women of reproductive age in Nigeria, using the recent NDHS data conducted in 2018. Generally, the study found that modern contraceptive use in Nigeria ranged from 0% to 75% across the country. The study also revealed a regional variation in modern contraceptive use among women of reproductive age. A higher proportion of modern contraceptive use was located in the Southern part of Nigeria, and a low proportion of modern contraceptive use was located in the Northern part of Nigeria. Specifically, a low proportion of modern contraceptive use was located in the Northern part of Nigeria, like Sokoto, Yobe, Zamfara, Borno, and Kebbi. Moreover, the country’s high proportion of modern contraceptive use was located in Kaduna, Adamawa, Ekiti, Rivers, and Federal Capital Territory.

The finding of this study is similar to what was observed in a previous study by Ajayi, Adeniyi and Akpan [21], which found that modern contraceptive use was high among women in some selected states in the Southern part of Nigeria. The regional variation in modern contraceptive use among Nigerian women could be attributed to differences in socio-cultural beliefs and practices. For example, in the Southern part of Nigeria, women are well-informed about contraceptive use and are more receptive to contraceptive usage compared to their Northern counterparts [21]. The finding suggests that there still exists some knowledge gap about the importance of modern contraceptive use among Nigerian women.

At the individual level, the study found that the likelihood of modern contraceptive use among women of reproductive age was high among those with secondary education and above compared to those with no education. This finding is similar to the finding of other previous studies [15, 22, 29] but disagrees with another study [30]. A plausible reason for this finding could be that women who have attained some level of education might have been educated about contraception use, options and the possible benefits of using modern forms of contraception, enhancing their likelihood of using such contraceptives [31]. Another explanation for this finding could be that the long duration of higher educational attainment may encourage women to use modern contraceptives to delay any unintended pregnancy until completion of education or after gaining employment [31]. Hence, making highly educated women more likely to use modern contraceptives.

In line with previous findings [16, 32], this study found that women from the Yoruba ethnic group were more likely to use modern contraceptives than those from the Hausa ethnic group. Women from the Hausa ethnic group may frown upon modern contraceptive use, hence reducing their likelihood to use modern contraceptives [16]. Another possible reason could be that, since the Hausa ethnic group permits polygamous marriages, women from this ethnic group would want to give birth to more children to be appreciated by their husbands [16].

Women who had four or more children were more likely to use modern contraceptives compared to those with no children. Similar findings were observed in other previous studies [4, 15, 21, 29, 30]. A plausible reason for this finding could be that women who have four or more children would have given birth and would not want to continue giving birth, increasing their likelihood of using modern contraceptives to prevent any unintended pregnancy [4, 30].

Similar to the findings of other previous studies [29, 32, 33], this study found that women exposed to mass media had a higher likelihood of using modern contraceptives than women who were not exposed to mass media. A possible reason for this finding could be that women exposed to mass media might have been as well exposed to adequate contraceptive education and would prefer using one to prevent unintended pregnancy or sexually transmitted infections [32].

Similar to the findings of other previous studies [4, 29, 30], this study found that women who were 35 years and above were less likely to use modern contraceptives compared to those aged 15–24. A possible reason for this finding could be that women aged 35 and above are married and would want to give birth compared to their younger counterparts, making them less likely to use modern contraceptives [4, 30].

Another important finding in this study was that married women were less likely to use modern contraceptives compared to never-married women. This is analogous to the findings of other previous studies [9, 34]. This study, however, contradicts the finding of a previous study [35]. An acceptable explanation for this finding could be that married women may be expected to give birth after marriage, hence, reducing their likelihood to use contraceptives [9]. Another reason for this finding could be that unmarried women may want to prevent any disgrace that comes with giving birth out of wedlock, which increase their likelihood of using modern contraceptives [35].

As found in previous studies [16, 32], in terms of religion, this study also found that Muslim women were less likely to use modern contraceptives compared to Christians. A potential explanation for this finding could be that women who practice Islam are at the disadvantage of using modern contraceptives due to their religious beliefs compared to their Christian counterparts [31].

At the household/community level, women from the richest household were more likely to use modern contraceptives than those residing in the poorest household. Similar observations were made in other previous studies [4, 22, 29]. Various pathways could explain this finding. Firstly, women from rich homes can afford modern contraceptives, increasing their likelihood of using modern contraceptives compared to the poor [22, 35]. Second, rich women are more empowered to make informed decisions about sexual engagements, increasing their likelihood of using modern contraceptives [4, 29].

Similar to the findings of other previous studies [29, 30], the study found that women residing in a community with medium knowledge of the modern contraceptive method and those with high literacy levels [29, 30] were more likely to use modern contraceptives. It is argued that women living in communities with a high literacy level have been well-educated about the importance, sources, and ways of using modern contraceptives, increasing their likelihood of using modern contraceptives [30]. It could also be that those women living in communities with a low literacy level have been brainwashed about the misconceptions and myths that accompany the use of modern contraceptives. Hence, they are less likely to use such contraceptives [14, 29].

Finally, our study also found that women residing in the Northwest region were less likely to use modern contraceptives than those in the Northcentral region. This finding corroborates a recent study by Oyinlola, and Bamiwuye [36], which concluded that there is a regional variation in the use of modern contraceptives among women in Nigeria. A possible reason for this finding could be that women residing in the Northwest region are less educated, reducing their likelihood to use modern contraceptives [22]. Another reason for this finding could be that since women residing in the Northwest region are less educated, they are more likely to be poor; hence, they are less likely to use modern contraceptives [22], or maybe the women of reproductive age residing in these regions desire more children [37].

### Strengths and limitations

Our study has several strengths. First, the use of nationally representative data boosts the capacity of our findings to be generalized to women in Nigeria. Additionally, the use of geographical information systems (GIS) in the analysis of the spatial distribution enabled us to identify the hotspots of modern contraceptive use in Nigeria, which is a major contribution to the literature on modern contraceptive use in Nigeria. Moreover, identifying the modern contraceptives’ hotspots would benefit both program designers and implementers in their design on context-specific and population-targeted interventions to enhance modern contraceptive use. Nevertheless, the study was not without some limitations. A major limitation to this study was that the data used was from a cross-sectional survey, limiting us from establishing causality. The data was self-reported, making it highly susceptible to recall bias and social desirability bias. The dataset does not consider the non-representativeness of the respondents at lower levels of geographic organization (e.g., the state), and there is a possibility of displacement of latitude and longitude on the interpretation of the mapping results.

### Policy and practical implications

Findings from our study has implications for policy and practice. For policy, the findings call for policymakers’ urgent attention to devise pragmatic ways to bridge this knowledge gap, which will help alleviate unwanted pregnancies and clandestine abortions. Women should be encouraged to pursue higher education to increase the likelihood of using modern contraceptives and have control over the number of children they desire. Family planning education programs should be broadcasted through mass media platforms, which will help improve modern contraceptive utilization among women of reproductive age in Nigeria. These programs should target communities with low literacy levels. Women in the Northwest region of Nigeria should be given critical attention in family planning education programs. More efforts should be invested in providing family planning education and services to women without fertility intentions in these regions, as this will further increase the rate of modern contraceptive use among women of reproductive age in Nigeria.

## Conclusion

The study found that modern contraceptive use in Nigeria ranged from 0% to 75% across the country, with regional variations. The study found that a higher proportion of modern contraceptive use was located in the Southern part of Nigeria (e.g., Kaduna, Adamawa, Ekiti, Rivers, and Federal Capital Territory), and a low proportion of modern contraceptive use was located in the Northern part of Nigeria (e.g., Sokoto, Yobe, Zamfara, Borno, and Kebbi). To further improve modern contraceptive use among women of reproductive age in Nigeria, factors identified in this study should be given maximum attention.

### Ethical approval

Since the authors of this manuscript did not collect the data, we sought permission from the MEASURE DHS website, access to the dataset and spatial geo-point of Nigeria was provided after our intent for the request was assessed and approved on the 5th of April 2021 with a clause of having at least one author from Nigeria if the data will be used for publication. The DHS surveys are ethically accepted by the ORC Macro Inc. Ethics Committee and the Ethics Boards of partner organizations in different countries, such as the Ministries of Health. The interviewed women gave either written or verbal consent during each of the surveys and followed Helsinki’s declaration of ethical principles.
